# Evaluation of enhanced external counterpulsation therapy for nonarteritic anterior ischemic optic neuropathy

**DOI:** 10.1186/s12886-020-01509-7

**Published:** 2020-06-17

**Authors:** Lixia Lin, Wenhui Zhu, Nan Ma, Xiaofeng Lin, Hui Yang

**Affiliations:** 1 Zhongshan Ophthalmic Center, State Key Laboratory of Ophthalmology, Sun Yat-sen University, Guangzhou, 510060 China; 2 Department of Ophthalmology, The First Affiliated Hospital of Sun Yat-sen University, Guangzhou, China

**Keywords:** Nonarteritic anterior ischemic optic neuropathy, Enhanced external counterpulsation, Visual acuity, Visual field

## Abstract

**Background:**

To explore the effects of enhanced external counterpulsation (EECP) and its underlying influencing factors in nonarteritic anterior ischemic optic neuropathy (NAION) patients.

**Methods:**

Patients at Zhongshan Ophthalmic Center with recent-onset (< 8 weeks) NAION were retrospectively recruited. The patients had decided whether or not they would undergo EECP treatment, and the patients who declined were included in the control group. The effectiveness of EECP was evaluated by comparing the visual function and fellow eye involvement in patients with and without EECP treatment.

**Results:**

In total, 61 patients (76 eyes) were included. Twenty-nine patients (37 eyes) underwent EECP treatment, while 32 patients (39 eyes) were included in the control group. Mean time from NAION onset to EECP initiation was 27.59 ± 16.70 days. In the EECP group, the mean EECP duration was 31.57 ± 18.45 days. EECP was well tolerated by all patients. However, there was no significant difference in visual function between the EECP and control groups. Furthermore, there was no evidence of the effectiveness of EECP in the subgroup analysis of patients with different systemic health conditions. Among the 42 patients with monocular NAION, the sequential attack rate was comparable between the EECP (27.78%) and control (25.00%) groups.

**Conclusion:**

This study is the first nonrandomized controlled study to evaluate the effectiveness of EECP in NAION patients. Unfortunately, we failed to demonstrate the effectiveness of EECP in NAION at the 6-month follow-up. Any further application of EECP in NAION patients should be cautious.

## Background

Nonarteritic anterior ischemic optic neuropathy (NAION) is an acute vision-threatening neurologic disorder commonly seen in elderly people [1]. Typically, the vision loss is usually monocular but, in some cases, binocular vision loss occurs in sequence or even simultaneously [2]. The precise pathogenesis of NAION is unclear. A variety of potential NAION risk factors can cause regional hypoxemia-related disc edema, resulting in axon death [3]. Some researchers have suggested that NAION is caused by circulatory insufficiency of the short posterior ciliary arteries, leading to laminar infarction of the optic nerve [4]. Several interventions have been proposed based on this theory [3–5]. The prognosis of NAION is still very poor, although the visual acuity improvement is relatively common. Moreover, no treatment has been shown to be substantially effective for vision function restoration or prevention of fellow eye involvement [5, 6].

Enhanced external counterpulsation (EECP) involves a noninvasive mechanical circulatory support system and it is used as an adjunctive therapy in patients with ischemic vascular diseases [7]. Using inflatable cuffs around the lower limbs, EECP increases the aortic diastolic pressure and cardiac output by increasing the venous volume return to the heart, leading to increased reperfusion of the affected arteries. Considering the “ischemic nature” of NAION, we previously investigated the use of EECP in NAION patients, which showed promising short-term results in improvement of visual acuity [8]. However, to the best of our knowledge, no controlled study with long-term follow-up and detailed visual function results has been reported regarding the effectiveness of EECP in NAION patients. Hence, this study was conducted to further explore the long-term benefit of EECP and its underlying influencing factors in NAION patients, by comparing the clinical outcomes of patients treated with and without EECP.

## Methods

The study was approved by the Ethics Committee of Zhongshan Ophthalmic Center. The study adhered to the Declaration of Helsinki. Patients with recent-onset (< 8 weeks) NAION were retrospectively recruited from the neuro-ophthalmology department of Zhongshan Ophthalmic Center. NAION diagnosis was made according to the Ischemic Optic Neuropathy Decompression Trial (IONDT) diagnostic criteria [9].

Detailed data from the patients’ medical records were collected, including medical history, medication usage history, and ophthalmic examination results: best-corrected vision acuity (BCVA), funduscopy, visual field (VF), fundus fluorescein angiography (FFA), visual evoked potential (VEP), and optical coherence tomography (OCT) results. Other routine assessments, including a blood pressure assessment, comprehensive blood tests, brain magnetic resonance imaging (MRI), and carotid artery color ultrasound imaging, had been conducted for each NAION patient (to exclude other diagnoses). Data from these assessments were collected retrospectively from the medical records to evaluate systemic health conditions (i.e., hypertension, diabetes mellitus, hypercholesterolemia, and ischemic stroke and etc.).

After the potential benefits and safety of EECP was explained by an ophthalmologist in detail, the patients had voluntarily decided whether or not to undergo EECP. The patients who had not elected to undergo EECP were included in the control group. After providing informed signed consent for EECP treatment, EECP patients attended the EECP Center at the First Affiliated Hospital of Sun Yat-sen University (Guangzhou, China), where they were scheduled to attend 1-h daily EECP sessions for up to 2 months. Patients were allowed to withdraw from treatment at any time. If any adverse event was reported, the clinician could also discontinue EECP.

All the patients, including EECP patients, were prescribed vitamin B12 and citicoline for at least 2 months. Patients with systemic risk factors were advised to continue receiving medical care from related specialists at the same time. Biweekly neuro-ophthalmology visits were scheduled until 2 months after NAION onset, followed by bimonthly visits for another 4 months. Detailed ophthalmic examinations were conducted at every visit, except for VEP and FFA assessments, which were only conducted in the first visit.

The exclusion criteria were as follows: suspicion of toxic, infiltrative, hereditary, or compressive optic neuropathy, arteritis, or any other prior ocular condition that might affect visual acuity or the visual field; lacking 6-month follow-up data; and EECP treatment < 7 days sessions in the EECP group; EECP contradictions (history of bypass surgery, heart attack, heart failure, aortic disease, aortic insufficiency, congestive heart disease, abdominal aortic aneurysm, venous thrombosis, frequent arrhythmia, or severe peripheral vascular disease [10]).

Detailed EECP procedures have been described previously, and the same protocol was used for all the patients [8, 11]. The EECP device (Shuangshan EECP-MCI, Guangzhou, China) inflates and deflates leg cuffs (using an air compressor) in a sequence synchronized to the patient’s cardiac cycle. The compression pressure of the cuffs was 0.035–0.040 mPa/cm.

### Statistical methods

The data analyses were performed using SPSS 19.0 (SPSS, Chicago, IL, USA). To evaluate the effectiveness of EECP on NAION, the eyes with NAION were divided into the EECP group and control group according to whether the patients chose EECP treatment or not. For patients with sequential attacks, only the eye with recent-onset NAION was included. Baseline differences between groups were compared with the independent-samples t-test and chi-square test. The independent t-test was also employed to evaluate between-group differences in treatment outcomes at 2 and 6 months, including the change in BCVA (logMAR), change in VF (mean deviation, MD) and retinal nerve fiber layer thickness (RNFLT). According to IONDT, the appropriate therapeutic window for NAION might be within 2 weeks after onset [9]. Accordingly, the EECP group was further divided into two groups: EECP within 2 weeks and EECP after 2 weeks. One-way analysis of variance (ANOVA) was employed to evaluate the differences between the control group, EECP group with EECP initiated within 2 weeks and EECP group with EECP initiated after 2 weeks. The level of significance was set at 0.05 (two tailed).

## Results

A total of 61 patients with 76 eyes from August 2015 to August 2018 were retrospectively included in this study. There were 38 (62.30%) males and 23 (37.70%) females. The mean age was 53.28 ± 9.40. Twenty-nine patients (37 eyes) underwent EECP treatment, leaving 32 participants (39 eyes) enrolled in the control group. In the EECP group, the mean time from NAION onset to EECP initiation was 27.59 ± 13.56 days and the mean EECP duration was 31.57 ± 18.45 days. No adverse events were reported.

There was no significant difference between the two groups regarding baseline characteristics (including the systemic risk factors). Detailed information is summarized in Table 1.

**Table 1:** Baseline characteristics and risk factors of EECP and control groups

	EECP	Control	*P* value
Age	53.81 ± 10.34	54.31 ± 9.41	0.419 ^a^
Sex, M:F	23:14	23:16	0.776 ^b^
Systemic risk factors			
Hypertension	17 (45.95%)	15 (38.46%)	0.509 ^b^
Diabetes mellitus	12 (32.43%)	19 (48.72%)	0.149 ^b^
Hypercholesterolemia	13 (35.14%)	13 (33.33%)	0.869 ^b^
Ischemic lesion on brain MRI	7 (18.92%)	10 (25.64%)	0.482 ^b^
Carotid artery plaque	8 (21.62%)	6 (15.38%)	0.483 ^b^
High blood viscosity	6 (16.22%)	4 (10.26%)	0.668 ^b^
Apnea	3 (8.11%)	4 (10.26%)	0.942 ^b^
Anemia	2 (5.41%)	4 (10.26%)	0.720 ^b^
Hepatitis B	2 (5.41%)	4 (10.26%)	0.720 ^b^
Hypoxia	3 (8.11%)	0 (0.00%)	0.220 ^b^
Alcoholism	2 (5.41%)	5 (12.82%)	0.471 ^b^
Smoking	0 (0.00%)	3 (7.69%)	0.258 ^b^
No of eyes attacked	37 (100.00%)	39 (100.00%)	

*EECP* enhanced external counterpulsation

^a^Independent t-test

^b^Chi-square test

### Effect of EECP on visual function

As shown in Table 2, there was no significant difference in the visual function between the EECP and control groups, regardless of follow-up time. The mean BCVA at presentation was 0.74 ± 0.62 and 0.99 ± 1.14 (*P* = 0.095) in the EECP and control groups, respectively. At month 2, the corresponding BCVA changes were 0.02 ± 0.44 and − 0.16 ± 0.59 (*P* = 0.261). At month 6, the corresponding BCVA changes were 0.04 ± 0.51 and − 0.02 ± 0.35 (*P* = 0.264). The mean MD at presentation was − 20.93 ± 8.77 and − 19.26 ± 9.81 (*P* = 0.239) in the EECP and control groups, respectively. At month 2, the corresponding MD changes were 0.04 ± 3.15 and 1.57 ± 4.38 (*P* = 0.287). At month 6, the corresponding MD changes were 0.27 ± 3.15 and 2.28 ± 4.80 (*P* = 0.443). The mean RNFLT values at presentation were 166.63 ± 82.01 and 175.61 ± 71.51 (*P* = 0.140) in the EECP and control groups, respectively; the corresponding values at month 6 were 65.10 ± 21.42 and 65.63 ± 19.43 (*P* = 0.600).

**Table 2:** Visual function differences among groups at baseline and 2- and 6-month follow-up

	Total	Control group	EECP group			*P* value ^a^	*P* value ^b^
				EECP initiated within 2 weeks	EECP initiated after 2 weeks	(t-test)	(ANOVA)
Eyes (n)	76	39	37	8	29		
BCVA							
Baseline	0.87 ± 0.93	0.99 ± 1.14	0.74 ± 0.62	0.66 ± 0.48	0.76 ± 0.65	0.095	0.492
Month 2	0.79 ± 0.72	0.82 ± 0.68	0.76 ± 0.76	0.80 ± 0.52	0.75 ± 0.83	0.954	0.926
Change (2–0)	−0.07 ± 0.52	−0.16 ± 0.59	0.02 ± 0.44	0.15 ± 0.25	−0.01 ± 0.47	0.261	0.211
Month 6	0.75 ± 0.61	0.72 ± 0.51	0.77 ± 0.72	0.83 ± 0.92	0.67 ± 0.62	0.174	0.145
Change (6–0)	0.01 ± 0.43	− 0.02 ± 0.35	0.04 ± 0.51	0.21 ± 0.32	− 0.05 ± 0.41	0.264	0.054
MD							
Baseline	−20.09 ± 9.27	−19.26 ± 9.81	−20.93 ± 8.77	−18.97 ± 7.07	−21.48 ± 9.24	0.239	0.602
Month 2	− 19.28 ± 9.05	−17.69 ± 9.41	− 20.88 ± 8.50	−18.76 ± 8.74	− 21.77 ± 8.49	0.459	0.248
Change (2–0)	0.81 ± 3.86	1.57 ± 4.38	0.04 ± 3.15	0.22 ± 2.48	− 0.01 ± 3.35	0.287	0.244
Month 6	−19.52 ± 9.09	−19.21 ± 9.89	−20.27 ± 7.24	−16.75 ± 15.91	−20.37 ± 5.71	0.096	0.798
Change (6–0)	1.30 ± 4.15	2.28 ± 4.80	0.27 ± 3.15	1.37 ± 5.98	0.07 ± 4.15	0.443	0.290
RNFLT							
Baseline	170.99 ± 76.65	175.61 ± 71.51	166.63 ± 82.01	155.42 ± 69.87	169.68 ± 85.65	0.140	0.801
Month 2	77.76 ± 29.40	81.44 ± 25.66	73.23 ± 33.48	82.00 ± 59.11	71.27 ± 27.31	0.835	0.511
Month 6	65.35 ± 20.24	65.63 ± 19.43	65.10 ± 21.42	57.67 ± 16.20	66.34 ± 22.30	0.600	0.796

*EECP* enhanced external counterpulsation, *BCVA* best-corrected visual acuity in LogMAR, *MD* mean deviation, *RNFLT* retinal nerve fiber layer thickness

^a^Independent t-test comparing visual function between the EECP and control groups

^b^One way ANOVA comparing the control group, EECP group with EECP initiated within 2 weeks and EECP group with EECP initiated after 2 weeks

Eight eyes underwent EECP within 2 weeks after NAION onset and 29 eyes initiated EECP after 2 weeks. No significant differences were observed by comparing these two groups with the control group (Table 2).

Additionally, there was no evidence of the effectiveness of EECP in the subgroup analysis of patients with different systemic health risk factors (hypertension, diabetes mellitus, hypercholesterolemia, and ischemic stroke) (Table 3).

**Table 3:** Comparisons of the visual function of the EECP and control groups by systemic health condition

	Hypertension		Diabetes mellitus		Hypercholesterolemia		Ischemic stroke	
	Yes	No	Yes	No	Yes	No	Yes	No
No. of eyes	32	44	31	45	26	50	17	59
EECP/Control (n)	17/15	20/24	12/19	25/20	13/13	24/26	7/10	30/29
BCVA								
Baseline	0.019^a^	0.921	0.065	0.625	0.626	0.086	0.059	0.102
	0.99 ± 0.70 vs 1.17 ± 1.07							
Month 2	0.885	0.650	0.917	0.826	0.178	0.661	0.035^a^	0.777
							0.62 ± 0.26 vs 0.95 ± 0.46	
Change (2–0)	0.122	0.800	0.087	0.511	0.759	0.308	0.204	0.265
Month 6	0.651	0.091	0.069	0.911	0.063	0.195	0.096	0.051
Change (6–0)	0.250	0.274	0.065	0.847	0.778	0.244	0.163	0.089
MD								
Baseline	0.063	0.901	0.153	0.797	0.394	0.453	0.849	0.134
Month 2	0.038^a^	0.422	0.106	0.662	0.814	0.474	0.650	0.425
	−21.88 ± 7.02 vs −19.18 ± 10.55							
Change (2–0)	0.490	0.139	0.758	0.096	0.128	0.799	0.240	0.155
Month 6	0.069	0.620	0.031^a^	0.885	0.744	0.056	0.096	0.200
			−20.98 ± 5.79 vs −18.92 ± 10.58					
Change (6–0)	0.609	0.911	0.202	0.244	0.425	0.715	0.748	0.460
RNFLT								
Baseline	0.910	0.013^a^	0.083	0.346	0.914	0.074	0.217	0.072
		170.63 ± 80.25 vs 189.38 ± 82.58						
Month 2	0.385	0.137	0.329	0.295	0.162	0.666	0.295	0.689
Month 6	0.827	0.089	0.417	0.616	0.054	0.715	0.448	0.096

*EECP* enhanced external counterpulsation, *BCVA* best-corrected visual acuity in LogMAR, *MD* mean deviation, *RNFLT* retinal nerve fiber layer thickness

Independent t-test was conducted to compare visual function between EECP and control groups

^a^Detailed parameter descriptions (EECP group vs control group) are displayed for cases involving a significant difference

### Effect of EECP on prevention of fellow eye NAION involvement

No recurrent attack was observed in any patients. Among the 42 patients with monocular NAION, five (27.28%) in the EECP group and six (25.00%) in the control group (*P* = 0.879) experienced fellow eye involvement during the 6-month follow-up. The mean intervals between the two attacks were 83.40 ± 41.57 and 70.00 ± 52.38 (*P* = 0.305), respectively (Table 4).

**Table 4:** Comparison of NAION patterns between the two groups

Pattern	EECP	Control	*P* value
Simultaneous binocular attack	3 (6)	3 (6)	
Sequential attacks (only the recent-onset eye was included)	8 (8)	5 (5)	
Presented with monocular attack	18 (23)	24 (28)	
Sequential fellow eye attack	5 (10)	6 (10)^a^	0.879 ^b^
Interval between two attacks (d)	83.40 ± 41.57	70.00 ± 52.38	0.305 ^c^
Fellow eye intact	13 (13)	18 (18)	
Total	29 (37)	32 (39)	

*NAION* non-arteritic anterior ischemic optic neuropathy, *EECP* enhanced external counterpulsation

Data show the no. of subjects (no. of eyes)

^a^Two subjects were lost to follow-up after fellow eye attack

^b^ Chi-square test, which was conducted with the no. of subjects

^c^ Independent t-test

## Discussion

Despite years of clinical and basic science studies, the exact mechanism of NAION is still unknown [3]. Briefly, the ultimate axonal degeneration and retinal ganglion cell apoptosis are comprehensive results of vascular ischemia (which causes anterior optic nerve edema), edema in a crowded disc (which causes compartment syndrome), and a subsequent cascade of vasogenic and cytotoxic factors [4, 12]. All these events ultimately lead to axonal degeneration and retinal ganglion cell apoptosis. Accordingly, potential therapeutic interventions targeting different parts of the pathogenic mechanism have been developed.

Histopathological and electron microscopic studies have shown that initial ischemia in the anterior optic nerve head is probably triggered by acute hypoperfusion of the vascular network originating from the short posterior ciliary arteries [13]. Previous studies have reported that not only can EECP accelerate reperfusion of the poorly perfused cerebral microvasculature and retinal artery, increase the blood flow velocity of the ophthalmic artery, but it can also improve endothelial function and collateral angiogenesis [14–16]. Additionally, EECP is thought to be highly safe and well-tolerated in selected patients [7, 17]. Therefore, EECP was expected to be a safe and effective intervention in ocular ischemic diseases. Previously, we reported in 16 NAION patients that visual acuity had improved from 0.92 to 0.40 (LogMAR) after EECP treatment (1 h daily for 12 days) [8]. However, the study did not have a control group. Therefore, the current controlled study with detailed examinations and a long follow-up period was deemed necessary to further evaluate the safety and long-term benefits of EECP treatment for NAION.

Unfortunately, this study demonstrated no obvious long-term benefit of EECP treatment in NAION patients with NAION onset < 8 weeks, regarding visual function and risk of fellow eye involvement. During the treatment period, which was no less than the treatment durations in the previous EECP studies reporting promising results regarding angina [7, 17–19], the safety of EECP was confirmed.

IONDT reported that around 40% of NAION patients might spontaneously regain 3-line visual acuity, which might explain the paradox involving the current study and our previous study, as the improvement in our previous study was probably not attributable to EECP [20]. There are few other studies on the effectiveness of EECP in NAION patients. One such study reported significant visual acuity improvement after EECP treatment in patients with carotid artery stenosis-related ocular ischemic diseases, including ischemic optic neuropathy (which includes more conditions than NAION), retinal artery occlusion, and ocular ischemic syndrome [16]. The study did not include a control group and the curative rate for the ischemic optic neuropathy subgroup was not reported. Thus, the effectiveness of EECP of NAION has not been proven.

Among the therapies tested in NAION patients, aspirin and anticoagulants have been acted on thrombosis and both vasopressors and vasodilators have been proposed to be useful for regulating vasodynamic factors [6, 21–24]. Although they all aimed to resolve the initial triggers of disc edema, there was no visual function improvement from any of the treatments. Considering the results of the current study, we speculate that interventions targeting the pathophysiological factors that are relevant prior to disc edema might not be helpful for improving visual function. Once disc edema is triggered, a destructive closed loop may be formed by a cascade of events, including compartment syndrome with axonal and capillary compression, increased ischemia, increased release of cytotoxic factors, and vasogenic and cytotoxic disc edema [4, 25, 26]. Future interventions should pay more attention to disrupting this loop. Furthermore, novel NAION animal models have recently emerged, which will help researchers to evaluate new drugs [27].

As stated by Newman et al., one of the most important approaches regarding NAION management is to reduce the risk of fellow eye involvement [6]. To protect against fellow eye involvement, potential etiological factors should be corrected first [6]. As mentioned above, EECP can accelerate reperfusion and increase the blood flow velocity of the ophthalmic artery, which might improve the vascular circulation of the fellow eye and theoretically prevent fellow eye involvement. However, EECP did not show any protective effect regarding fellow eye involvement in this study. The rate of sequential attack during the 6-month follow-up period was relatively high (25–28%) compared to that in other studies (15–20%) [28, 29]. This might be explained by the fact that patients without fellow eye involvement can easily be lost to follow-up after stabilization of the visual acuity in the first eye. As a result, the rate of fellow eye involvement identified in this retrospective study might be much higher than the actual rate. As optic nerves are the nerve endings of the cerebrum, many consider NAION and intracranial cerebrovascular ischemic disease to be similarly related [30]. Consequently, long-term aspirin use has also been recommended by some practitioners as a secondary prevention method, despite the controversy regarding the current evidence [22]. A large randomized controlled trial is necessary to provide more credible evidence.

As a retrospective study, some limitations were inevitable in the current study. As the patients had chosen whether or not to undergo EECP treatment freely, this may have led to unrecognized bias, even though there were no significant differences regarding baseline characteristics (including the risk factors) between the two groups. Although there is no definite therapeutic window for NAION treatment, many studies have specified a 2-week window [9, 31, 32]. Most patients in current study had exceeded 2-week window at presentation. In order to rule out the time of treatment as a confounding factor, comparison was made to observe the difference between the eight eyes underwent EECP within the 2-week therapeutic window and the 29 underwent EECP after this window. Even though no differences were observed, the issue remains to be a limitation should be emphasized. After all, effort was devoted to improving the current frustrating situation regarding NAION treatment, but no significant effects were observed after EECP treatment.

## Conclusion

This study is the first controlled study with a long-term follow-up period to evaluate the effectiveness of EECP treatment in NAION patients and to assess its prevention effect on fellow eye involvement. By comparing the groups with and without EECP treatment, we failed to demonstrate the effectiveness of EECP in NAION patients with onset < 8 weeks. A randomized controlled trial with prompt EECP treatment is recommended, until then, any further application of EECP in NAION patients should be cautious.

## Data Availability

The datasets generated during and analyzed during the current study are not publicly available due to patient privacy but are available from the corresponding author on reasonable request.

## Abbreviations

NAION: Nonarteritic anterior ischemic optic neuropathy
EECP: Enhanced external counterpulsation
IONDT: Ischemic Optic Neuropathy Decompression Trial
BCVA: Best-corrected vision acuity
VF: Visual field
FFA: Fundus fluorescein angiography
VEP: Visual evoked potential
OCT: Optical coherence tomography
MRI: Magnetic resonance imaging
